# Decoding the saline-alkaline tolerance nexus in soybean: a dual-method evaluation model coupled with co-expression networks identifies core regulatory genes

**DOI:** 10.3389/fpls.2025.1699904

**Published:** 2025-11-27

**Authors:** Liu Fei, Bai Xionghui, Li Mengjiao, Hao Aijing, Xing Baolong

**Affiliations:** 1 High Latitude Crops Institute to Shanxi Academy, Shanxi Agricultural University, Datong, China; 2 Maize Research Institute, Shanxi Agricultural University, Xinzhou, China; 3 Social Service Department, Shanxi Agricultural University, Jinzhong, China

**Keywords:** saline-alkali, soybean, ranking model, WGCNA, candidate gene

## Abstract

**Introduction:**

Soybean (Glycine max) growth is severely restricted by the high salt content in saline-alkali soils, resulting in substantial declines in both yield and quality. Enhancing soybean’s tolerance to saline-alkali stress holds significant economic and ecological importance. However, current research on the regulatory mechanisms of soybean’s response to such stress, especially when integrating physiological traits with transcriptomic analyses, remains inadequate.

**Methods and Results:**

In this study, seven physiological indicators of soybean cultivars showed significant differences between saline-alkali and normal conditions, and there were correlations among their rates of change. The salt tolerance rankings obtained by principal component analysis combined with the membership function value method were verified to be reliable by the technique for order preference by similarity to an ideal solution (TOPSIS). Transcriptome analysis identified 4,582 differentially expressed genes (DEGs), 39 of which were differentially expressed in all tissues and varieties. Weighted gene coexpression network analysis (WGCNA) determined the gene modules related to physiological traits.

**Discussion:**

Through comprehensive analysis, 13 core candidate genes were found, providing a basis for elucidating the molecular mechanisms of soybean’s adaptation to saline-alkali conditions.

## 1 Introduction

As a major abiotic stress, soil salinization poses a critical threat to global agriculture, affecting approximately 20% of the arable land. This includes arid inland regions, where salt accumulates due to evaporation, and coastal zones impacted by seawater intrusion (Chu et al., 2024). Soybean (Glycine max) serves as a typical glycophyte and demonstrates pronounced sensitivity to saline-alkaline conditions. During germination, elevated osmotic pressure in such soils impedes water uptake, resulting in reduced germination rates (Zhang et al., 2023; Zhou et al., 2023). Root development remains severely constrained throughout the plant life cycle, thereby limiting nutrient acquisition and leading to yield losses exceeding 50% (Ren et al., 2023). As a crucial source of high-quality plant protein and oil, soybean provides essential amino acids and unsaturated fatty acids, establishing its indispensable role in human nutrition. Given the accelerating global population and increasing food demand, soybean cultivation in saline-alkaline regions has become a strategic approach for enhancing productivity and ensuring food security.

Elucidating the mechanisms underlying soybean tolerance to saline-alkali stress is essential for breeding high-tolerance cultivars and improving yield in affected regions. Saline-alkali stress markedly inhibits seed germination and plant growth, reduces photosynthetic efficiency, and diminishes yield (Feng et al., 2021. Ullah et al., 2019). Physiologically, exposure to such stress induces excessive accumulation of reactive oxygen species (ROS), including superoxide anions (O2^−^) and hydrogen peroxide (H2O2), resulting in oxidative damage. To mitigate this, antioxidant defense systems are activated, involving enzymes, such as superoxide dismutase (SOD), peroxidase (POD), and catalase (CAT). SOD catalyzes the conversion of O2^−^ into H_2_O_2_, which is subsequently decomposed into water and oxygen by POD and CAT, thereby alleviating oxidative injury. Numerous studies have confirmed that salt-tolerant soybean cultivars generally exhibit enhanced antioxidant enzyme activities under saline-alkali conditions (Basit et al., 2023; Zhang et al., 2023, 2019; Sabljic et al., 2020). Simultaneously, the accumulation of osmotic adjustment substances, including proline, soluble sugars, and betaine, counteracts the elevated soil osmotic pressure, which impairs root water uptake and leads to cellular dehydration. These compounds reduce cellular osmotic potential, facilitate water absorption, and improve drought and salt tolerance (Cui et al., 2022; Gai et al., 2020). For instance, the proline content in soybean leaves significantly increases under saline-alkali stress, contributing to adaptation to high-salinity environments. While physiological traits reflect soybean responses to saline-alkali stress, the associated regulatory mechanisms remain poorly understood. With advances in genomics and transcriptomics, increasing attention has been directed towards the genetic basis of stress tolerance. Various transcription factors (TFs) have been implicated in this regulatory network. Families such as WRKY, NAC, and bZIP are known to regulate gene expression related to stress adaptation (Shu et al., 2022. Yang et al., 2022). These TFs bind to the promoter regions of target genes, either activating or repressing transcription, to modulate tolerance. Additionally, signaling pathways, particularly those involving abscisic acid (ABA) and calcium ions, play critical roles in the perception and transduction of saline-alkali stress signals (Zhang et al., 2022).

Current evaluation systems for soybean salt-alkali tolerance predominantly rely on single biochemical indicators (e.g., proline content) or unidimensional statistical methods, such as principal component analysis (PCA) (Hu et al., 2024). Although these approaches facilitate preliminary screening, they often oversimplify the complex, dynamic interactions across multiple physiological responses and organs. In multi - attribute evaluation, single methods have limitations. To improve result reliability and accuracy, this study uses a dual - validation approach, applying the PCA + membership function method and TOPSIS simultaneously. The former can handle data complexity and uncertainty, while the latter focuses on alternative comparison. Their mutual verification allows multi - perspective evaluation and compensates for single - method drawbacks. Under salt stress, antioxidant enzymes, including SOD, CAT, and POD, along with osmolytes (e.g., soluble proteins), exhibit coordinated, tissue-specific activities. However, PCA-based models frequently fail to capture the spatiotemporal complexity. Although the membership function method has demonstrated promise for comprehensive trait ranking in crops such as maize and wheat, its integration with decision-making algorithms remains largely unexplored in soybean research (Liu et al., 2015). Notably, recent legume studies employing the Technique for Order Preference by Similarity to Ideal Solution (TOPSIS) combined with Manhattan distance metrics have primarily focused on germination indices, overlooking whole-plant physiological dynamics. This highlights a critical gap in the evaluation framework for perennial crops. Despite its potential to identify stress-responsive candidates, transcriptome-based gene discovery suffers from two key methodological limitations. First, most studies prioritize extreme phenotypes, disregarding intermediate genotypes that may harbor distinct regulatory architectures. This “binary classification” contradicts the field data, which indicate that 68% of soybean germplasms exhibit a gradient, rather than a dichotomous, salt-alkali response. Second, prevailing tissue-specific analyses, typically limited to shoots or roots, fail to account for the systemic nature of salt adaptation. Multi-tissue metabolomic profiling has revealed that 73% of salt-responsive metabolites display inverse accumulation patterns between aerial and subterranean organs, suggesting that single-tissue transcriptomics may overlook the key hub genes responsible for maintaining whole-plant homeostasis. Moreover, only 12% of published soybean transcriptome studies validate candidate genes through physiological evaluation models, underscoring a persistent disconnect between molecular findings and phenotypic relevance (Liu et al., 2023. Xu et al., 2023).

In this study, a novel evaluation framework was developed by integrating the membership function method with TOPSIS to rank soybean cultivars based on their dynamic physiological responses. The indicator weights were objectively determined using the entropy weight method, thereby improving the robustness of saline-alkaline tolerance assessments. To link phenotypic evaluation with molecular mechanisms, multi-tissue transcriptome profiling (above- and below-ground organs) was conducted on cultivars with contrasting tolerance rankings. The core gene modules associated with physiological traits were identified through differential expression analysis and WGCNA, enabling the selection of candidate genes via cross-group and network intersections. This study aimed to (1) establish a robust multi-indicator model for evaluating soybean saline-alkaline tolerance and (2) elucidate the genetic basis of tolerance through integrative physiological and transcriptomic analyses. These results provide a practical tool for cultivar screening and offer novel gene targets for molecular breeding.

## 2 Materials and methods

### 2.1 Plant materials and experimental design

Nine soybean cultivars, including Tongdou 15 (TD15), Qinong 52 (QN52), Jindou 46 (JD46), Jindou 49 (JD49), Qinong 54 (QN54), Tongqin 1 (TQ1), Tongdou 5 (TD5), Jindou 1 (JD1), and Changdou 32 (CD32), with diverse genetic backgrounds were selected from the national germplasm collection to encompass a broad spectrum of responses to salt-alkali stress. Seed samples were obtained from the National Genebank and stored under optimal conditions prior to experimentation. Seeds of each cultivar were surface sterilized using 1% sodium hypochlorite for 10 min, followed by thorough rinsing with distilled water. Germination was conducted on moist filter paper in Petri dishes at 25 °C in complete darkness for 48 h. After germination, the seedlings were transplanted into plastic pots (20 cm diameter) filled with a 3:1 (v/v) mixture of loam and sand. The pots were maintained in a greenhouse under controlled conditions: a 14-hour photoperiod, day/night temperatures of 25/18°C, and 65% relative humidity.2.2 Correlation analysis of physiological indices before and after Saline-Alkaline Stress

### 2.2 Saline-alkaline stress treatment

When the seedlings reached the V3 growth stage, they were divided into two groups: a control group and a stress group.

Control Group: The plants in the control group were irrigated with Hoagland solution (pH 6.5, electrical conductivity (EC) = 1.2 dS/m) throughout the experiment.

Stress Group: The plants in the stress group were treated with a salt-alkali solution containing 150 mM NaCl and 50 mM NaHCO3 (pH 8.5, EC = 15 dS/m) for 14 d. This treatment was designed to simulate the typical salt-alkali soil conditions in soybean-growing areas.

### 2.3 Determination methods for different physiological indicators

After 14 d of stress treatment, leaf samples were collected from both control and stress groups of each cultivar to measure seven physiological indicators. All tests were conducted with three replicates.

#### 2.3.1 Superoxide dismutase activity

SOD activity was determined based on its capacity to inhibit photochemical reduction of nitroblue tetrazolium (NBT). Leaf tissue (0.5 g) was homogenized in 5 mL of 50 mM phosphate buffer (pH 7.8) containing 1% polyvinylpyrrolidone (PVP). The homogenate was centrifuged at 12,000 × g for 20 min at 4°C, and the resulting supernatant was used for the assay. One unit of SOD activity was defined as the amount of enzyme required to inhibit NBT reduction by 50%.

#### 2.3.2 Catalase activity

CAT activity was measured by monitoring the decomposition of hydrogen peroxide (H2O2) at 240 nm. Leaf tissue (0.5 g) was homogenized in 5 mL of 50 mM phosphate buffer (pH 7.0) containing 1 mM ethylenediaminetetraacetic acid (EDTA) and 1% PVP. The homogenate was centrifuged at 12,000 × g for 20 min at 4 °C, and the supernatant was used for the assay. The CAT activity was then expressed as units per milligram of protein (U/mg protein).

#### 2.3.3 Malondialdehyde content

MDA content was determined using the thiobarbituric acid (TBA) method. Leaf tissue (0.5 g) was homogenized in 5 mL of 10% trichloroacetic acid (TCA), and the homogenate was centrifuged at 10,000 × g for 15 min. An aliquot of the supernatant was mixed with an equal volume of 0.6% TBA in 10% TCA and incubated in a boiling water bath for 15 min. After cooling, absorbance was measured at 450, 532, and 600 nm. The MDA content was calculated using a standard formula.

#### 2.3.4 Peroxidase activity

The POD activity was measured by monitoring the oxidation of guaiacol at 470 nm. Leaf tissue (0.5 g) was homogenized in 5 mL of 50 mM phosphate buffer (pH 7.0) containing 1 mM EDTA and 1% PVP. The homogenate was centrifuged at 12,000 × g for 20 min at 4°C, and the supernatant was used for the assay. POD activity was calculated based on the rate of change in absorbance per minute.

#### 2.3.5 Soluble protein content

SP content was determined using the Coomassie Brilliant Blue G-250 method. Leaf tissue (0.5 g) was homogenized in 5 mL of 50 mM phosphate buffer (pH 7.0), and the homogenate was centrifuged at 10,000 × g for 15 min. The resulting supernatant was used for the assay with bovine serum albumin (BSA) as the standard.

#### 2.3.6 Proline content

Pro content was determined using the acid ninhydrin method. Leaf tissue (0.5 g) was homogenized in 5 mL of 3% sulfosalicylic acid, and the homogenate was centrifuged at 10,000 × g for 10 min. An aliquot of the supernatant was mixed with equal volumes of glacial acetic acid and acid ninhydrin reagent, and incubated in a boiling water bath for 30 min. After cooling, absorbance was measured at 520 nm. The Pro content was calculated using a standard curve.

#### 2.3.7 Antioxidant capacity

AOC was measured using a 2,2-diphenyl-1-picrylhydrazyl (DPPH) radical scavenging assay. Leaf tissue (0.5 g) was homogenized in 5 mL of 80% methanol, and the homogenate was centrifuged at 10,000 × g for 15 min. An aliquot of the supernatant was mixed with DPPH solution and incubated in the dark for 30 min. The absorbance was measured at 517 nm. AOC was expressed as the percentage of DPPH radical-scavenging activity.

### 2.4 Evaluation of salt - alkali tolerance using membership function value method

The change rates of the seven physiological indicators for each cultivar were standardized to eliminate the effects of different units and scales. The standardized values ${\text{X}}_{\text{ij}}\;$ for the i-th cultivar (i = 1, 2,…, and 9) and the j-th indicator (j = 1, 2,…, and 7) were calculated as follows:

For positive-effect indicators (e.g., SOD, CAT, POD, SP, and AOC): ${\text{X}}_{\text{ij}}=\frac{{\text{x}}_{\text{ij}}-\min({\text{x}}_{\text{j}})}{\max({\text{x}}_{\text{ij}})-\min({\text{x}}_{\text{j}})}$. For negative-effect indicators (e.g., MDA and Pro): ${\text{X}}_{\text{ij}}=\frac{\max({\text{x}}_{\text{j}})-{\text{x}}_{\text{ij}}}{\max({\text{x}}_{\text{ij}})-\text{min}({\text{x}}_{\text{j}})}$, where ${\text{X}}_{\text{ij}}\;$ is the original change rate value, and min( ${\text{x}}_{\text{j}}$) and max( ${\text{x}}_{\text{j}}$) are the minimum and maximum values of the j-th indicator among all cultivars, respectively. The membership function value ${\text{U}}_{\text{ij}}$ of the i-th cultivar for the j-th indicator was calculated using the standardized value ${\text{X}}_{\text{ij}}$: ${\text{U}}_{\text{ij}}={\text{X}}_{\text{ij}}$. The comprehensive membership function value ${\text{U}}_{\text{i}}$ of the i-th cultivar was calculated as the average of the membership function values of all seven indicators: ${\text{U}}_{\text{i}}=\frac{1}{7}\sum_{\text{j}=1}^{7}{\text{U}}_{\text{ij}}$. The larger the ${\text{U}}_{\text{i}}$ value, the stronger the salt-alkali tolerance of the i-th cultivar.

### 2.5 Validation of salt-alkali tolerance ranking using TOPSIS method

A standardized change rate matrix $\text{X}={\left({\text{X}}_{\text{ij}}\right)}_{9*7}$, derived using the membership function value method, was employed as the decision matrix. The positive ideal solution A+ and the negative ideal solution A− were determined as follows. The positive ideal solution ${\text{A}}^{+}=({\text{X}}_{1}^{+},{\text{ X}}_{2}^{+},\;\cdots ,{\text{ X}}_{7}^{+})$ is a 7-dimensional vector, where each component ${\text{X}}_{\text{j}}^{+}(\text{j}=1,\;2,\;\cdots ,\;7)$ represents the maximum value among the standardized values ${\text{X}}_{\text{ij}}$ for the j-th indicator across all nine cultivars, that is, ${\text{X}}_{\text{j}}^{+}={\text{max}}_{1\leq \text{i}\leq 9}\{{\text{X}}_{\text{ij}}\}$. Similarly, the negative ideal solution ${\text{A}}^{-}=({\text{X}}_{1}^{-},{\text{ X}}_{2}^{-},\;\cdots ,{\text{ X}}_{7}^{-})$ consists of components ${\text{X}}_{\text{j}}^{-}(\text{j}=1,\;2,\;\cdots ,\;7)$, each representing the minimum value among the ${\text{X}}_{\text{ij}}$ values for the j-th indicator, expressed as ${\text{X}}_{\text{j}}^{-}={\text{min}}_{1\leq \text{i}\leq 9}\{{\text{X}}_{\text{ij}}\}$. Here, i denotes the cultivar (i = 1 to 9), and j denotes the indicator (j = 1 to 7). ${\text{X}}_{\text{ij}}$ is the standardized value of the i-th cultivar for the j- th indicator. The Euclidean distances ${\text{D}}_{\text{i}}^{+}$ and ${\text{D}}_{\text{i}}^{-}$ of the i-th cultivar from the positive and negative ideal solutions, respectively, were calculated: ${\text{D}}_{\text{i}}^{+}=\sqrt{\sum_{\text{j}=1}^{7}{\left({\text{X}}_{\text{ij}}-{\text{X}}_{\text{j}}^{+}\right)}^{2}}$ and ${\text{D}}_{\text{i}}^{-}=\sqrt{\sum_{\text{j}=1}^{7}{\left({\text{X}}_{\text{ij}}-{\text{X}}_{\text{j}}^{-}\right)}^{2}}$. The relative closeness ${\text{C}}_{\text{i}}$ of the i-th cultivar to the positive ideal solution was then computed as: ${\text{C}}_{\text{i}}=\frac{{\text{D}}_{\text{i}}^{-}}{{\text{D}}_{\text{i}}^{+}+{\text{D}}_{\text{i}}^{-}}$. A higher ${\text{C}}_{\text{i}}$ value indicates stronger salt-alkali tolerance. The ranking of the nine soybean cultivars based on ${\text{C}}_{\text{i}}$ values was used to validate the ranking obtained via the membership function value method.

### 2.6 RNA extraction, illumina sequencing, and data analysis

Total RNA was extracted from 24 samples using Trizol reagent (Invitrogen, Carlsbad, CA, USA) according to the manufacturer’s protocol. Following the quality and quantity assessments performed using a NanoDrop One spectrophotometer (Thermo Fisher Scientific, Waltham, MA, USA), the samples were submitted to Baimaike Biotechnology Co., Ltd. (Beijing, China) for cDNA library construction. RNA integrity was verified using an Agilent 2100 Bioanalyzer (Agilent Technologies, Palo Alto, CA, USA), with samples meeting the minimum RNA Integrity Number (RIN) threshold of 7 required for library preparation. The libraries were prepared using the TruSeq RNA sample preparation kit (Illumina RS-122-2101, Illumina, CA, USA) and sequenced on an Illumina HiSeq 2000 platform generating 150 bp paired-end reads. The raw sequencing data were deposited in the National Genomics Data Center (NGDC), Beijing Institute of Genomics, Chinese Academy of Sciences, under Project PRJCA011580, Genome Sequence Archive (GSA) accession CRA008043 (https://ngdc.cncb.ac.cn/gsub/submit/gsa/subCRA012317/finishedOverview; accessed 12 December 2024). Quality control was performed using FastQC and Trimmomatic software (Chen et al., 2014). The clean reads were obtained by removing adapters, poly-N sequences, and low-quality reads, followed by the alignment to the G. max reference genome (Wang et al., 2023) downloaded from Ensembl Plants (http://plants.ensembl.org/species.html; accessed 18 December 2024) using HISAT2 (Kim et al., 2019). Transcript expression levels were quantified as fragments per kilobase of transcript per million mapped reads (FPKM) using StringTie. Differential expression analysis was performed using DESeq R package. Genes with fold change >1.5 and false discovery rate (FDR) <0.05 were considered DEGs. The functional enrichment of DEGs was performed via the DAVID platform (https://davidbioinformatics.nih.gov; accessed 30 December 2024).

### 2.7 Weighted gene co-expression network analysis

The gene co-expression network was constructed using the WGCNA package in R following the official WGCNA tutorial. Initially, clustering analysis was performed after excluding samples exhibiting a low correlation or failing to cluster on the dendrogram. To satisfy the scale-free topology criterion, the soft-thresholding power was determined using the “pickSoftThreshold” function by selecting the parameter at which the scale-free topology fit index first approached 0.9. Subsequently, a correlation-based association between phenotypes and gene modules was calculated to generate an adjacency matrix, which was transformed into a topological overlap matrix (TOM) to construct the gene co-expression network. Gene modules were identified and clustered using the dynamic tree cut method based on module eigengenes (MEs), with modules exhibiting high similarity. In this study, the module similarity threshold was set to 0.25, the expression threshold to 2, and minimum module size to 30 genes.

### 2.8 Screening and functional annotation of hub genes

Functional annotation was performed via homology-based comparison of candidate gene sequences against public databases such as NCBI. Functional information from highly homologous genes was used to preliminarily infer candidate gene functions. Furthermore, multiple sequence analyses were performed. The cis-regulatory elements within the 2000 bp upstream promoter regions of candidate genes were analyzed using the PlantCARE database. The identification of these cis-acting elements facilitated the prediction of transcription factors potentially binding to them, thereby elucidating the transcriptional regulatory mechanisms governing candidate genes.

### 2.9 Data analysis

Raw data were obtained from three biological replicates. Prior to analysis, the data were subjected to rigorous quality control and preprocessing to remove obvious outliers. Differential analysis was conducted using the t-test, with the specific test type chosen based on the data distribution. Statistical significance was set at p < 0.05. Correlation analysis was performed using R package “corrplot”. For normally distributed continuous variables, Pearson’s correlation coefficient was employed, whereas Spearman’s rank correlation coefficient was applied to the non-normally distributed data. Significant correlations were determined at p < 0.01.

## 3 Results

### 3.1 Physiological indicator changes in soybean cultivars under saline-alkaline stress

**Figure 1:**
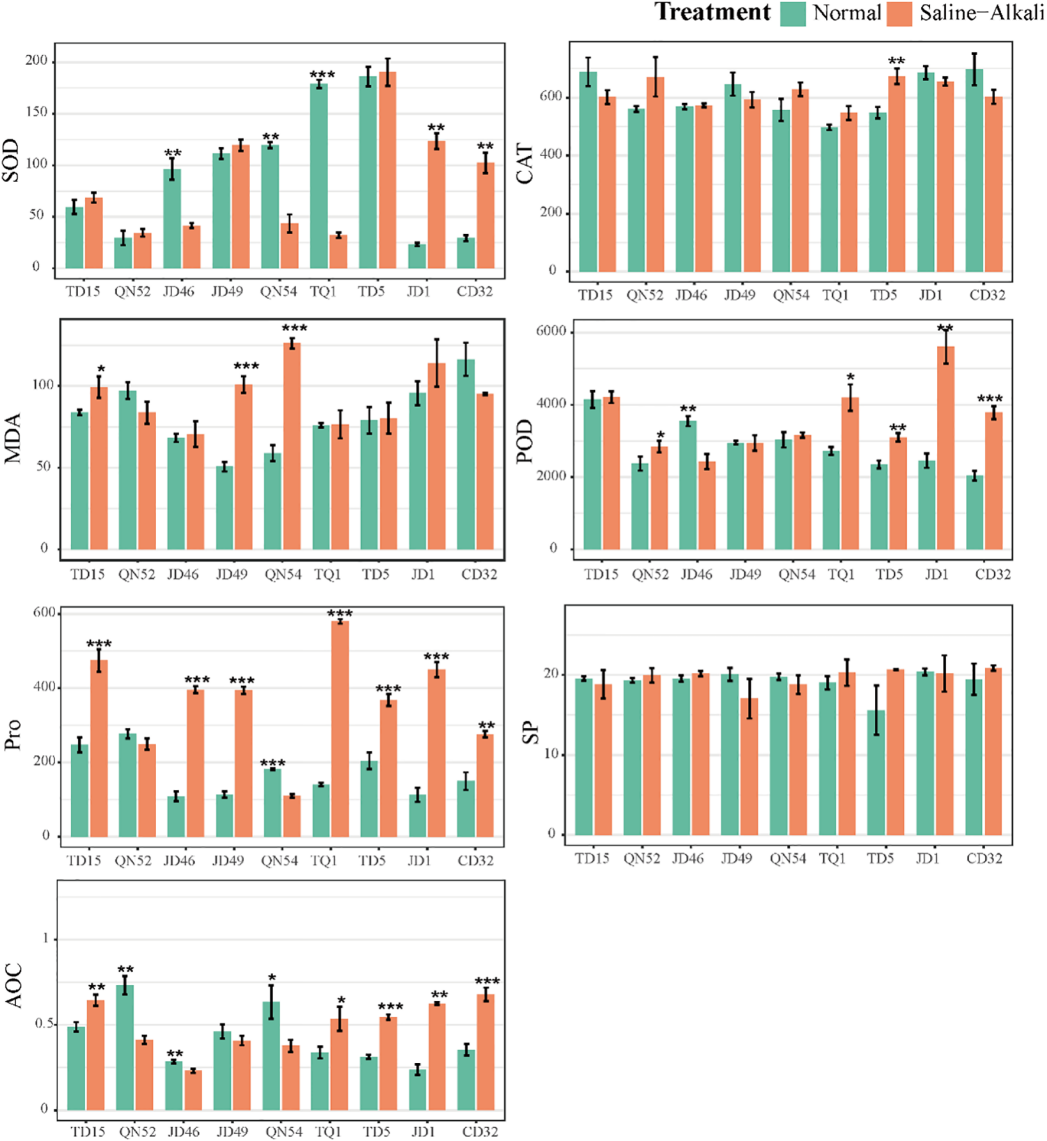
Bar chart showing differences in seven physiological indicators of nine varieties before and after salt stress. The x-axis represents the nine varieties, and the y-axis represents the values of the physiological indicators. The green bars represent the data before stress, and the orange bars represent the data after stress. Error bars represent standard errors. * indicates P < 0.05, ** indicates P < 0.01, and *** indicates P < 0.001.

The physiological profiling of nine soybean cultivars (TD15, QN52, JD46, JD49, QN54, TQ1, TD5, JD1, and CD32) under salt stress revealed the genotype-specific regulation of antioxidant defense and osmotic adjustment systems (**Figure 1**). The SP content remained stable across all cultivars (p > 0.05), indicating conserved proteostasis under saline conditions. CAT activity exhibited cultivar-dependent induction, with TD5 showing significant post-stress elevation, while others maintained baseline levels, consistent with CAT’s established role in the salt tolerance. Pro accumulation varied markedly, where QN54 displayed an unexpected reduction, QN52 showed no significant change, and other cultivars exhibited substantial increases, reflecting genotype-dependent Pro metabolism under ionic toxicity. The MDA levels, indicative of oxidative membrane damage, were significantly elevated in JD49 and QN54 but remained stable in the other cultivars. The SOD activity presented three distinct patterns, including significant suppression in TQ1, JD46, and QN54, enhancement in JD1 and CD32, and no significant change in others, which was consistent with the isoform-specific SOD regulation during salt adaptation. The POD activity followed contrasting trends, with QN32, TQ1, TD5, JD1, and CD32 exhibiting significant activation, whereas JD46 showed suppression, suggesting the cultivar-specific modulation of POD’s dual roles in reactive oxygen species scavenging and cell wall lignification.

### 3.2 Correlation analysis of physiological indices before and after saline-alkaline stress

**Figure 2:**
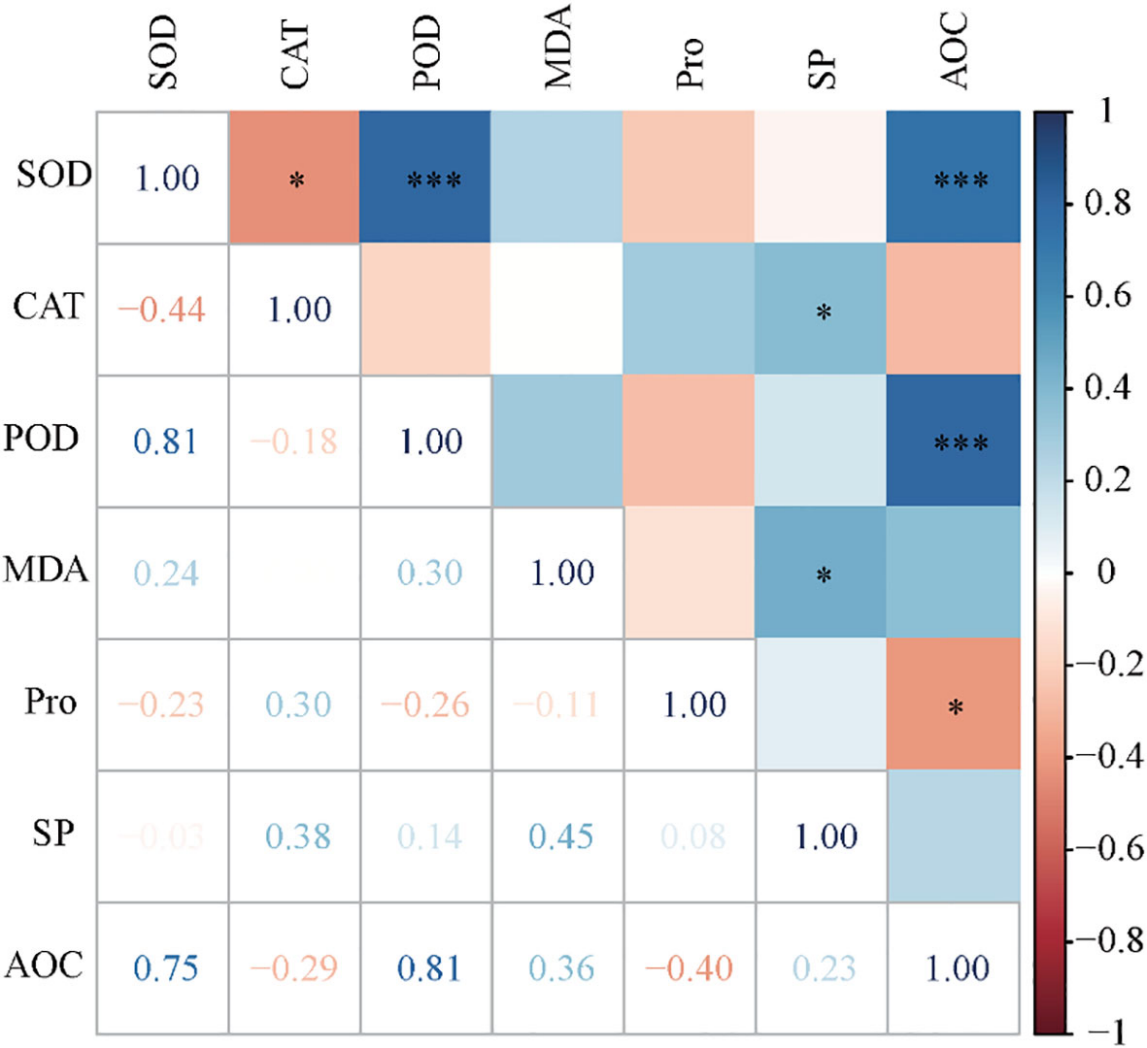
Heat map for correlation analysis of the change rates in seven physiological indicators before and after salt stress. * indicates P < 0.05, and *** indicates P < 0.01.

To elucidate the systemic interplay among stress-responsive traits, a correlation analysis of physiological indices revealed compartmentalized coordination patterns under salinity stress (**Figure 2**). Heatmap visualization identified significant negative correlations between CAT and SOD activities (r = −0.44, p < 0.05), as well as between the total AOC and Pro content (r = −0.40, p < 0.05). Conversely, SP was positively correlated with both MDA (r = +0.45, p < 0.05) and CAT (r = +0.38, p < 0.05), suggesting that protein stabilization may co-vary with oxidative damage and hydrogen peroxide detoxification. Notably, AOC was strongly positively correlated with SOD (r = +0.75, p < 0.001) and POD (r = +0.81, p < 0.001), which was accompanied by synergistic SOD-POD coordination (r = +0.81, p < 0.001). These pronounced correlations implied that SOD and POD jointly dominated the ROS scavenging efficacy, consistent with their complementary metalloenzyme functions within peroxisomal and mitochondrial compartments.

### 3.3 Ranking of salt tolerance of soybean varieties

A comprehensive salt tolerance evaluation was conducted using PCA integrated with the membership function method, wherein MDA and Pro were designated as negative indicators, and all other parameters as positive contributors. The composite scores ranked the cultivars in the descending order of tolerance: JD1 (0.76) > CD32 (0.64) > QN52 (0.49) > TQ1 (0.41) > TD5 (0.27) > TD15 (0.25) > JD46 (0.19) > JD49 (0.10) > QN54 (0.04). This ranking underscored JD1’s superior antioxidant coordination (notably SOD/POD synergy) and membrane stability (low MDA), which was in contrast to QN54 sensitivity and was marked by elevated oxidative damage and impaired osmoregulation. Validation via TOPSIS yielded largely consistent results, with only two rank reversals. QN52 and CD32 exchanged second and third positions (JD1 > QN52 > CD32 > TQ1 > TD5 > TD15 > JD46 > JD49 > QN54). The minimal discrepancy (Spearman’s ρ= 0.96, p < 0.001) confirmed the robustness of the methodology. The elevated TOPSIS ranking of QN52 likely reflected its pronounced CAT activity increase under stress, which was partially underweighted in the PCA membership analysis, whereas CD32’s slight descent aligned with TOPSIS’s sensitivity to its marginally lower SOD-driven antioxidant capacity compared to JD1.

### 3.4 Identification of DEGs in QN52 and JD49 under saline-alkaline stress

**Figure 3:**
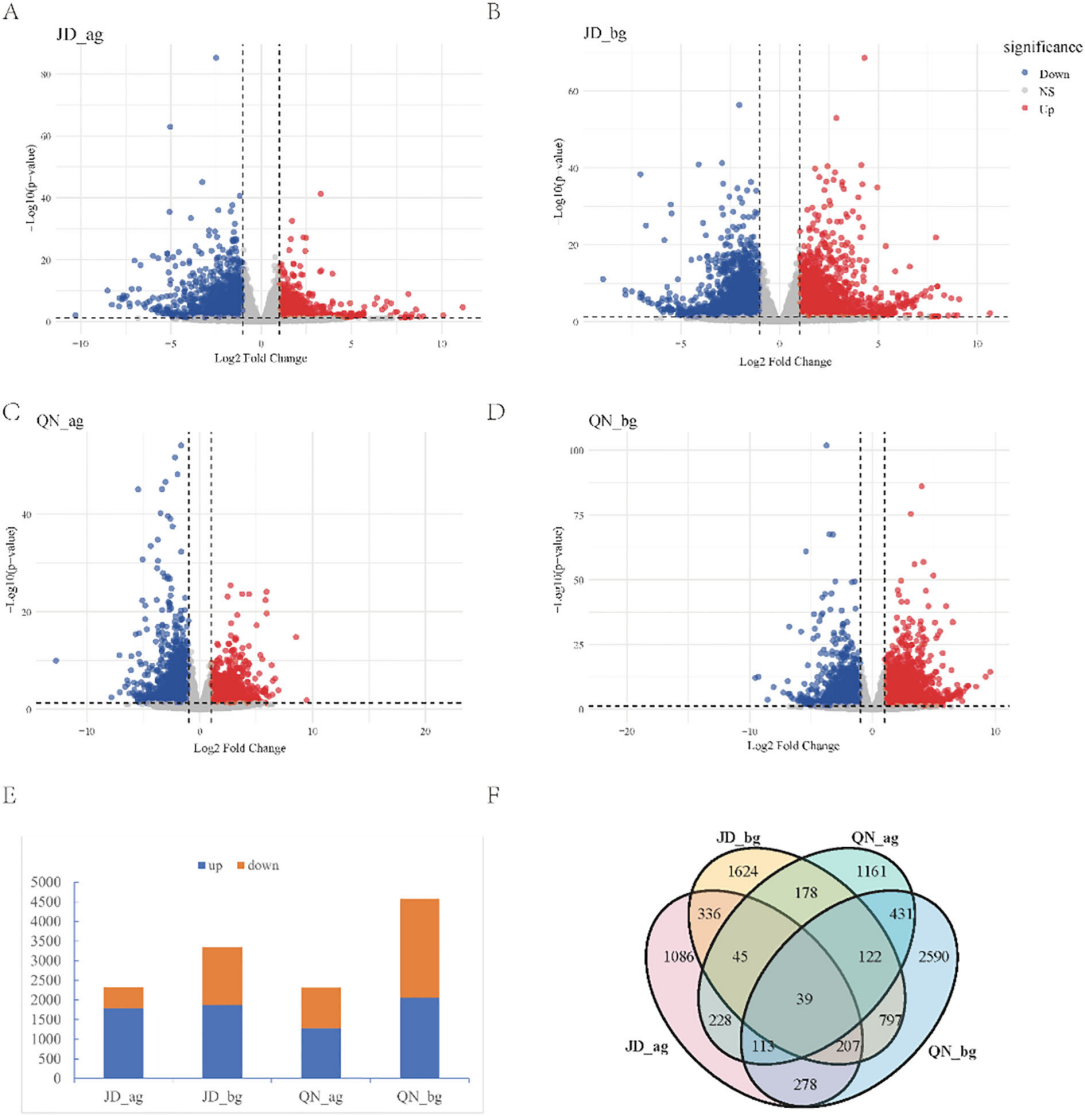
Analysis of differentially expressed genes (DEGs) before and after salt stress. **(A)** Volcano plot of DEGs in the above-ground tissues of JD49; **(B)** volcano plot of DEGs in the below-ground tissues of JD49; **(C)** volcano plot of DEGs in the above-ground tissues of Qinong 52; **(D)** volcano plot of DEGs in the below-ground tissues of Qinong 52. **(E)** Bar chart comparing the number of up-regulated and down-regulated genes across different tissues and varieties; the x-axis represents the combinations of varieties and tissues, and the y-axis indicates the number of DEGs. **(F)** Venn diagram showing the overlap of DEGs across all tissues and varieties; each circle represents a DEG set for a specific tissue–variety combination, and overlapping areas indicate shared genes.

Transcriptomic analysis provides a powerful approach for elucidating the molecular mechanisms underlying plant responses to saline-alkaline stress. This study focused on two soybean cultivars with contrasting salt tolerance: the moderately tolerant QN52 (ranked second) and the salt-sensitive JD49 (ranked seventh). Comprehensive transcriptome profiling was performed on both above-ground and under-ground tissues of these cultivars, identifying a total of 52,673 genes. In salt-sensitive JD49 (**Figure 3A**), gene expression patterns differed markedly from those of QN52. In the above-ground tissues, a dominant down-regulation trend was observed, with 1,792 genes down-regulated and 541 genes up-regulated (**Figure 3B**). In roots, although the gene expression also changed, a moderately activated state was evident, with 1,475 genes up-regulated and 1,877 down-regulated (**Figures 3C, D**). Conversely, in salt-tolerant QN52, the above-ground and under-ground tissues exhibited distinct transcriptional profiles. The above-ground tissues demonstrated 1,040 up-regulated and 1,278 down-regulated genes, while the roots demonstrated stronger transcriptional reprogramming, with 2,521 genes up-regulated and 2,061 down-regulated (**Figure 3E**). Venn diagram analysis identified 39 genes differentially expressed in both above- and below-ground tissues of the two cultivars (**Figure 3F**).

### 3.5 DEG enrichment analysis

**Figure 4:**
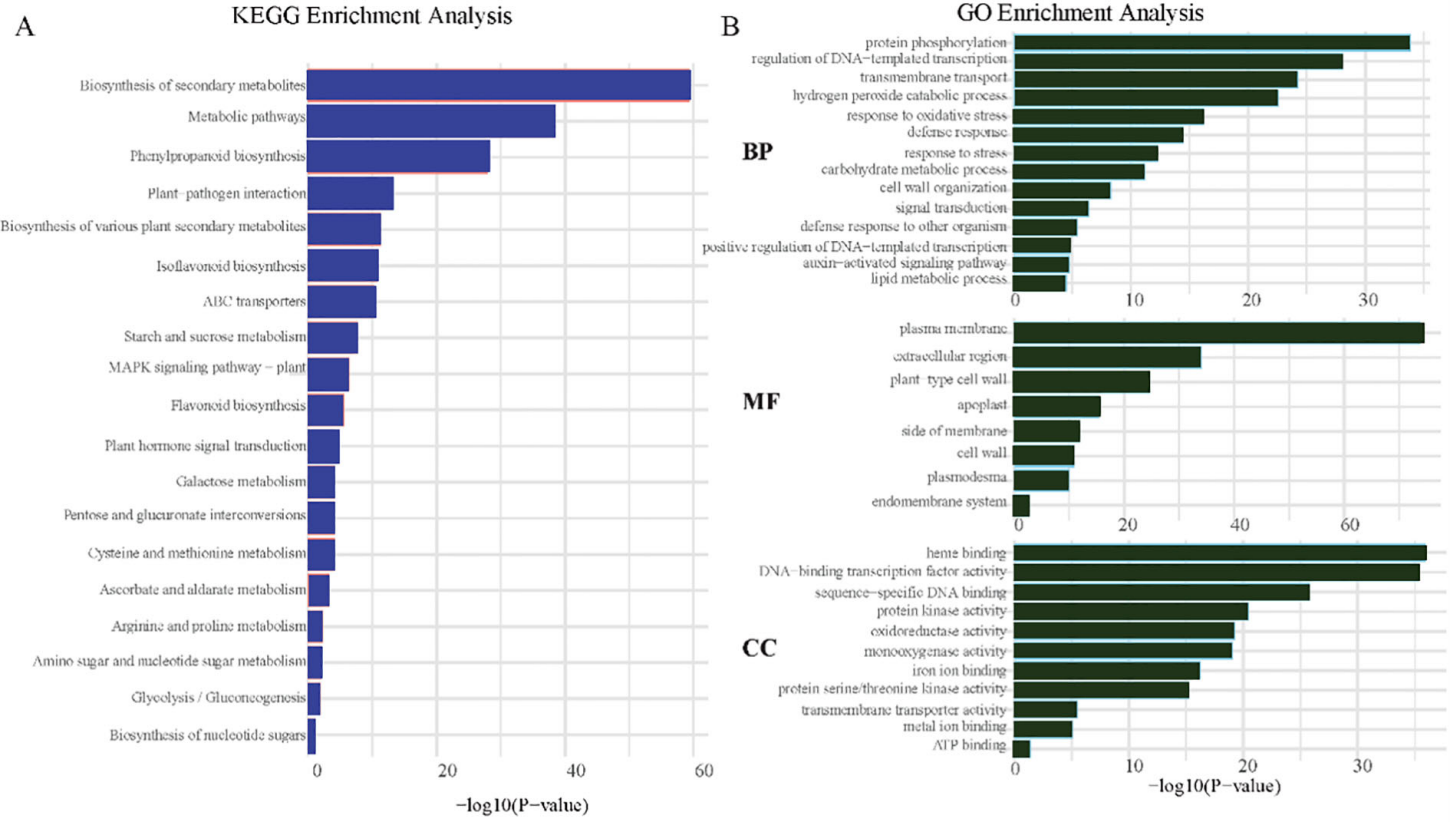
Enrichment analysis of differentially expressed genes (DEGs). The **(A)** displays the Kyoto Encyclopedia of Genes and Genomes (KEGG) enrichment analysis, highlighting the biological pathways significantly associated with DEGs. The **(B)** presents the Gene Ontology (GO) enrichment analysis, categorizing the functions of DEGs into three domains: molecular function, cellular component, and biological process.

Differential gene enrichment analysis was initially conducted using the KEGG pathway analysis (**Figure 4A**). The differentially expressed genes were predominantly enriched in three pathways: the biosynthesis of secondary metabolites essential for plant adaptation to environmental stresses, metabolic pathways reflecting significant impacts on the organism’s overall metabolic homeostasis, and phenylpropanoid biosynthesis involved in producing key secondary metabolites that contribute to plant defense and structural integrity. For GO enrichment analysis (**Figure 4B**), the genes in the Biological Process (BP) category serving as key post-translational modifications that regulate diverse cellular functions were predominantly enriched in protein phosphorylation and in the regulation of DNA-templated transcription, indicating stringent transcriptional control over differential gene expression. Within the Molecular Function (MF) category, significant enrichment was observed in the plasma membrane, extracellular region, and plant-type cell wall components, which were associated with cell integrity, plant-environment interactions, and mechanical support. Regarding the Cellular Component (CC) category, the genes were enriched in heme binding and vital for processes such as oxygen transport, DNA-binding transcription factor activity, and sequence-specific DNA binding, which play central roles in gene expression regulation.

### 3.6 Identification of hub-genes in response to salt stress

**Figure 5:**
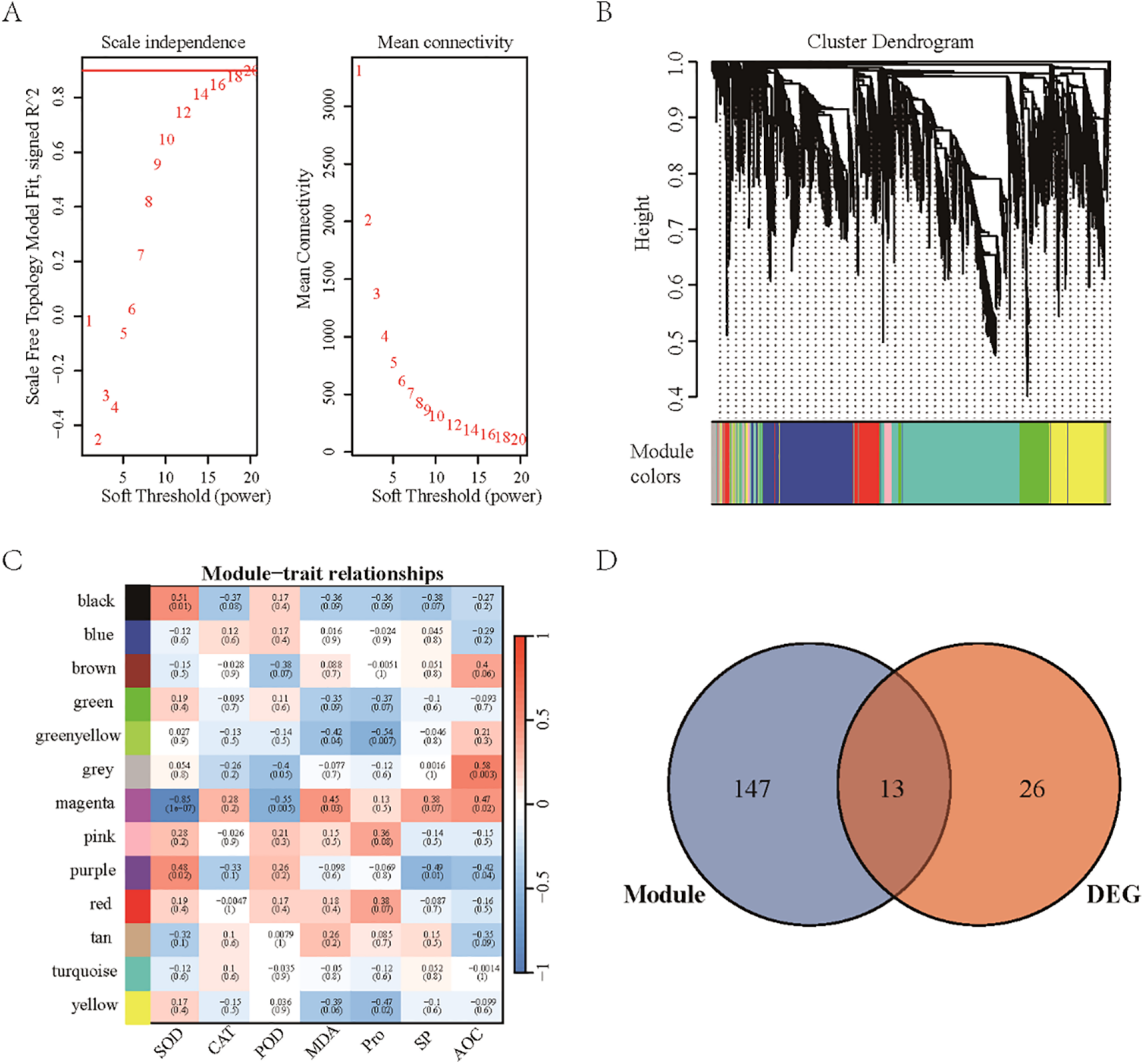
Results of weighted gene co-expression network analysis (WGCNA). **(A)** Soft threshold selection plot used to determine the optimal soft threshold power for constructing a scale-free network. **(B)** Module identification plot showing gene clustering into distinct modules based on expression patterns. **(C)** Heatmap of module–phenotype correlations illustrating the strength and direction of associations between each module and the measured phenotypes. **(D)** Venn diagram depicting the overlap between core genes from highly correlated modules and differentially expressed genes.

Weighted gene co-expression network analysis (WGCNA) was performed by integrating transcriptome-wide gene expression data with seven physiological indicators. Initially, the scale-free topology fit index and mean connectivity were evaluated for various soft threshold powers (**Figure 5A**), resulting in the selection of a soft threshold power of 20. At this threshold, the scale-free topology fit index approached 0.9, indicating that the constructed gene co-expression network approximated a scale-free topology, facilitating accurate identification of gene modules and their interrelationships. Using this soft threshold, all genes were clustered into 13 distinct modules (**Figure 5B**). Correlation analysis between these modules and physiological phenotypes revealed specific associations (**Figure 5C**), where the black module exhibited the strongest correlation with SOD activity; the magenta module correlated most closely with CAT, MDA, and SP contents, suggesting its involvement in antioxidant defense and protein metabolism; the purple module was primarily associated with POD content; the pink module showed the highest correlation with Pro content, potentially linked to osmotic adjustment under stress; and the grey module correlated most strongly with total AOC. To identify key genes within each module, the top 30 genes with the highest connectivity were selected as core genes. Subsequently, by intersecting these core genes with the 39 genes differentially expressed in both above- and below-ground tissues of the two cultivars (**Figure 5D**), 13 candidate genes were identified. These genes represent key regulators mediating the interaction between gene expression and physiological responses, providing valuable targets for future functional studies.

### 3.7 Gene annotation and promoter analysis of candidate genes

**Figure 6:**
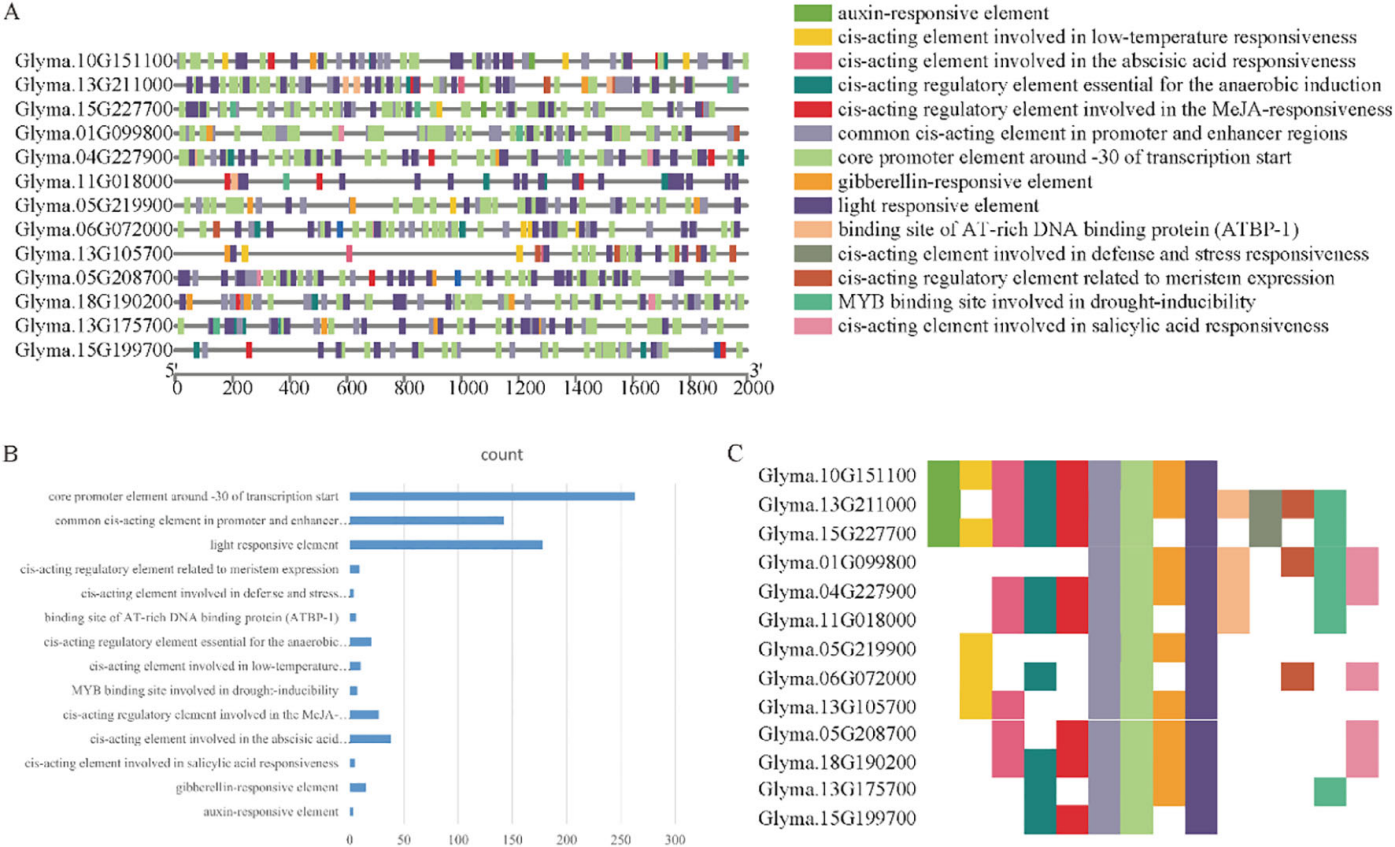
Promoter analysis of candidate genes. **(A)** Distribution of promoter elements across individual candidate genes. **(B)** Bar chart showing the frequencies of different promoter element types. **(C)** Overview of the presence of various promoter element categories across candidate genes using the same legend as in panel **(A)**.

To further elucidate the functions of the 13 candidate genes identified previously, gene annotation was performed (**Table 1**). Subsequently, promoter analysis was conducted to deepen functional insights. Fourteen types of cis-regulatory elements were identified across these genes, with a widespread distribution throughout their promoter regions (**Figure 6A**). The predominant elements included auxin-responsive elements, core promoter elements, light-responsive elements, growth-regulatory elements, and stress-responsive elements. Among these, the “core promoter around -30 of transcription start” was the most abundant (**Figure 6B**). This region is critical for transcription initiation and contains binding sites for RNA polymerase and associated transcription factors. Moreover, the “common cis-acting element in promoter and enhancer regions”, “core promoter element around -30 of transcription start”, and “light responsive element” were present in all 13 genes (**Figure 6C**). The ubiquitous presence of these elements suggests the coordinated regulation of these genes in response to diverse environmental and developmental cues, implicating their important roles in plant growth, development, and stress responses.

**Table 1:** Gene annotation of key candidate genes.

Gene_ID	Gene annotation
*Glyma.11G018000*	PROTEIN PHOSPHATASE 2C
*Glyma.13G175700*	SMALL HEAT-SHOCK PROTEIN HSP20 FAMILY
*Glyma.18G190200*	GLUTATHIONE S-TRANSFERASE, GST, SUPERFAMILY,
*Glyma.06G072000*	EXPRESSED PROTEIN
*Glyma.01G099800*	Glutamyl-gamma-semialdehyde dehydrogenase
*Glyma.13G105700*	HEAT STRESS TRANSCRIPTION FACTOR A-9
*Glyma.10G151100*	EXPRESSED PROTEIN
*Glyma.15G199700*	Adhesive plaque protein
*Glyma.15G227700*	EamA-like transporter family (EamA)
*Glyma.05G208700*	AQUAPORIN PIP1-4-RELATED
*Glyma.04G227900*	OXIDOREDUCTASE, 2OG-FE II OXYGENASE FAMILY PROTEIN
*Glyma.13G211000*	F-box associated interaction domain
*Glyma.05G219900*	NAD(P)-BINDING ROSSMANN-FOLD SUPERFAMILY PROTEIN

## 4 Discussion

This study conducted an in-depth analysis of the physiological responses of nine soybean cultivars under salt stress. The results demonstrated significant genotype-specific variations, providing important insights into the salt tolerance mechanisms of soybean and informing salt-tolerant breeding strategies. Cultivars TD5 and QN52 exhibited strong salt tolerance, which is closely associated with their physiological characteristics. Notably, CAT activity in TD5 increased significantly following salt stress, which is consistent with CAT’s established role in salt-tolerant genotypes. CAT effectively scavenges excess hydrogen peroxide, thereby mitigating oxidative damage. Previous studies have confirmed that enhanced CAT activity contributes to improved salt tolerance in various plant species. Although the Pro content in QN52 remained unchanged under salt stress, osmotic balance may be maintained through alternative pathways (Kucukoglu Topcu and Bhalerao, 2023). Additionally, both cultivars displayed low membrane lipid peroxidation levels and stable MDA content, indicating that preserved membrane integrity is conducive to maintaining normal cellular physiological function. Membrane lipid peroxidation can be recognized as a key indicator of cellular damage under stress, with stable MDA level ls supporting plant physiological stability during salt exposure (Kumar et al., 2023. Mahmud et al., 2023).

In contrast, the QN54 and JD49 cultivars were sensitive to salt stress. Pro accumulation in QN54 showed an abnormal decrease, likely due to the disruption of Pro metabolic pathways under ionic toxicity, which prevented effective osmotic adjustment. Previous studies have established the pivotal role of Pro in plant osmotic regulation, with insufficient accumulation compromising salt stress adaptation. The antioxidant enzyme systems in these cultivars were inhibited to varying extents. Specifically, the SOD activity in QN54 declined significantly, reducing the capacity for superoxide anion scavenging. MDA content increased markedly, indicating pronounced oxidative damage to cell membranes. This damage further compromised cellular physiological functions, increasing sensitivity to salt stress. It has been reported that diminished antioxidant enzyme activity can promote the ROS accumulation, exacerbating the membrane lipid peroxidation and thereby reducing salt tolerance (Zang et al., 2015).

Correlation analysis revealed complex coordination patterns among soybean physiological indicators under salt stress. A significant negative correlation between CAT and SOD activities was observed, suggesting a suggestive of functional complementarity and regulatory interplay among antioxidant enzymes to maintain ROS homeostasis. As the activity of one enzyme increases, the other may decrease to preserve the cellular ROS balance, which is consistent with previous findings. The negative correlation between total AOC and Pro content implies a trade-off in resource allocation between antioxidant defense and osmotic adjustment. The positive correlations of SP with MDA and CAT suggest that protein stability is closely linked to oxidative damage and hydrogen peroxide detoxification. The strong positive correlations between AOC, SOD, and POD, along with the synergistic interaction between SOD and POD, highlight their predominant roles in ROS scavenging. These metalloenzymes function complementarily within organelles, such as peroxisomes and mitochondria, jointly enhancing the ROS clearance efficiency. Previous studies have demonstrated that SOD and POD act synergistically in plant antioxidant defense, efficiently mitigating oxidative damage (Ron et al., 2022. Shah and Nahakpam, 2012; Faryal et al., 2022).

The PCA membership function method offers a robust and effective comprehensive approach for evaluating salt tolerance in soybean by integrating multiple physiological indicators. MDA and Pro were designated as negative indicators, whereas other parameters served as positive contributors, enabling an objective ranking of soybean varieties. The resulting rankings demonstrated strong concordance with those obtained using TOPSIS, with only minor positional discrepancies. This concordance confirmed the reliability of the PCA membership function method and underscored its high consistency with TOPSIS in salt tolerance assessments. Previous studies have indicated that comprehensive evaluation methods can provide more accurate assessments of plant salt tolerance, highlighting the broad applicability of both PCA-membership and TOPSIS approaches. The elevated TOPSIS ranking of QN52 likely reflected its unique CAT activity, which was more fully captured by this method. Conversely, the slight decline in CD32 ranking may be attributed to TOPSIS’s heightened sensitivity to its relatively lower SOD-driven antioxidant capacity.

For breeding strategies, it is recommended to prioritize salt-tolerant cultivars, such as TD5 and QN52, as parental lines for cross-breeding. Recombination and integration of their salt-tolerance genes are expected to facilitate the development of new cultivars with enhanced salt tolerance. Numerous studies have demonstrated that cross-breeding can combine superior traits from different varieties to improve salt tolerance in crops. Concurrently, molecular marker-assisted selection targeting gene loci associated with antioxidant enzyme activities and proline metabolism can enhance breeding efficiency and accuracy, thereby accelerating the development of salt-tolerant varieties. Relevant research has confirmed that marker-assisted selection can effectively screen plants harboring salt tolerance-related genes, serving as a powerful tool for salt-tolerant breeding (Hasan et al., 2021). Additionally, the application of exogenous substances such as EA has been shown to increase CAT activity and enhance antioxidant capacity in soybeans. This approach offers a novel avenue for improving salt tolerance and can be integrated into breeding programs to further enhance the salt tolerance of soybean cultivars (Ni et al., 2022).

Significant differences in gene expression patterns were observed between QN52 and JD49. In QN52, the gene expression changes in above-ground tissues were relatively balanced, indicating moderate transcriptional adjustment under saline-alkali stress with limited amplitude. In contrast, the roots exhibited pronounced transcriptional reprogramming, with extensive gene up- and down-regulation, suggesting a pivotal role for roots in adapting to the saline-alkali environment. Conversely, JD49 above-ground tissues displayed a marked down-regulation trend, implying severe inhibition of growth and metabolic processes and an impaired ability to activate defense mechanisms. Although root gene expression changes in JD49 indicated moderate activation, its overall salt tolerance and regulatory capacity remained limited. These observations are consistent with previous findings. For instance, Li et al. reported significant differences in gene expression patterns between above- and below-ground tissues in crop varieties with contrasting salt tolerance under saline-alkali stress (Li et al., 2023). Salt-tolerant varieties typically exhibit strong root gene regulation as an adaptive strategy. These results highlight that soybean varieties can employ distinct response strategies to saline-alkali stress, with salt-tolerant cultivars demonstrating enhanced root-based transcriptional regulation (Şekerci et al., 2024).

Differential gene enrichment analysis revealed that KEGG pathways significantly enriched for DEGs included secondary metabolite biosynthesis, metabolic pathways, and phenylpropanoid biosynthesis. Secondary metabolite biosynthesis plays a critical role in plant adaptation to environmental stress, and alterations in metabolic pathways have profound effects on the organism’s overall metabolic balance. Phenylpropanoid biosynthesis contributes to the synthesis of key secondary metabolites involved in plant defense and structural integrity, underscoring the activation of self-protection mechanisms under saline-alkali stress. Numerous studies have validated the importance of these pathways in plant responses to abiotic stresses. For example, Fang et al. reported significant up-regulation of genes in the secondary metabolite and phenylpropanoid biosynthesis pathways in rice subjected to saline-alkali stress, enhancing stress resistance (Fang et al., 2023). GO enrichment analysis revealed that within the Biological Process (BP) category, the genes were predominantly enriched in protein phosphorylation and DNA-templated transcriptional regulation, indicating that post-translational modifications and transcriptional control are pivotal for differential gene expression. In the Molecular Function (MF) category, enrichment related to the plasma membrane, extracellular region, and plant-type cell wall highlighted the importance of cell integrity, plant-environment interactions, and mechanical support. In the Cellular Component (CC) category, the enrichment of heme binding, DNA-binding transcription factor activity, and sequence-specific DNA binding underscored the fundamental mechanisms of gene expression regulation. Mechanistically, the secondary metabolite synthesis pathway regulates osmotic pressure by increasing the synthesis of substances such as proline. The products of the phenylpropanoid pathway can scavenge reactive oxygen species (ROS) and alleviate oxidative damage. Both pathways are also involved in signal transduction, activating salt - alkali tolerance - related genes. The enrichment analysis results in **Figure 4** show that the expression of these genes is up - regulated under salt - alkali stress. Additionally, lignin produced by the phenylpropanoid pathway reinforces the cell wall. Similar findings have been reported in other species. Jiang et al. demonstrated that enrichment of genes related to protein phosphorylation and transcriptional regulation was closely linked to maize adaptability to drought stress (Jiang et al., 2023).

In WGCNA, a soft-threshold power of 20 was selected to construct a gene co-expression network approximating a scale-free topology, which facilitated accurate identification of gene modules and their interrelationships. Thirteen distinct gene modules were identified, each of which exhibited specific correlations with the physiological indicators. For instance, the black module showed a strong association with SOD activity, suggesting that the genes within this module may play pivotal roles in SOD-related antioxidant defense mechanisms. Similarly, Wang et al. reported that in rice, particular gene modules were closely linked to antioxidant enzyme activities, underscoring the critical role of gene modules in plant antioxidant defense (Wang et al., 2022).

Annotation analysis of 13 candidate genes provided valuable insights into their potential functions. The glutathione S-transferase (GST) superfamily gene (Glyma.18G190200) played a critical role in plant defense against saline-alkali stress. GSTs catalyze the conjugation of glutathione to toxic by-products generated by oxidative stress, thereby protecting plant cells from damage. Numerous studies have confirmed the involvement of GSTs in plant stress resistance. For instance, Moons demonstrated that GSTs are up-regulated in response to various stresses, including oxidative and heavy metal stress, facilitating the maintenance of cellular redox homeostasis (Liu et al., 2015). The small heat-shock protein HSP20 family gene (Glyma.13G175700) functions as a molecular chaperone, preventing protein misfolding and aggregation under saline-alkali stress to preserve cellular function (Liu et al., 2015). The aquaporin PIP1–4 gene (Glyma.05G208700) regulates water permeability in plant cells, aiding adaptation to water-deficit conditions induced by saline-alkali soils (Lopes-Caitar et al., 2013). Promoter analysis revealed the widespread distribution of various cis-acting elements across the 13 candidate genes, including auxin-responsive elements, core promoter elements, light-responsive elements, growth-regulatory elements, and stress-responsive elements. Among these, the “core promoter around -30 of the transcription start site” was the most abundant, representing a critical site for transcription initiation due to its binding of RNA polymerase and associated factors. Furthermore, “common cis-acting elements in the promoter and enhancer regions”, “core promoter elements around -30 of the transcription start site”, and “light-responsive elements” were present in all 13 genes, suggesting coordinated regulation by multiple environmental and developmental signals. Similar patterns have been reported in other species. For example, Fleurat et al. observed comparable distributions of cis-acting elements in the promoter regions of Arabidopsis genes involved in stress responses, implying the coordinated regulatory mechanisms underlying plant stress adaptation.

## 5 Conclusions

This study integrated physiological index analysis with transcriptome sequencing to achieve significant advances in the evaluation of soybean salt tolerance and the identification of associated regulatory genes. Significant differences were observed in seven physiological indices among soybean varieties grown in saline-alkali versus normal soils. The correlated change rates of these indices indicated their interrelated roles in the salt-alkali stress response, providing a foundation for understanding soybean physiological mechanisms. A robust salt-tolerance ranking and validation model was established by combining PCA with the membership function method and TOPSIS, enabling efficient screening of salt-tolerant cultivars. Transcriptomic analysis identified a total of 4,582 genes were found to be differentially expressed, among which 39 genes were differentially expressed in all tissues and varieties. Enrichment analysis revealed that these genes predominantly participated in the stress response and metabolic regulation pathways, with the gene counts supporting the accuracy of the tolerance ranking. The WGCNA detected the modules highly correlated with physiological indicators, and the intersection with the 39 genes yielded 13 core candidate genes. Gene annotation and promoter analyses provided preliminary insights into their potential salt-tolerance regulatory mechanisms. These findings could lay the groundwork for future functional validation and molecular breeding efforts aimed at improving soybean yield in saline-alkali soils and enhancing food security.

## Data Availability

The original contributions presented in the study are included in the article/supplementary material. Further inquiries can be directed to the corresponding author/s.
